# Efficient production and characterization of melanin from *Thermothelomyces hinnuleus* SP1, isolated from the coal mines of Chhattisgarh, India

**DOI:** 10.3389/fmicb.2023.1320116

**Published:** 2024-01-12

**Authors:** Shalini Pandey, Vineet Meshram, Hany M. Yehia, Abdulhakeem Alzahrani, Nadeem Akhtar, Arunima Sur

**Affiliations:** ^1^Amity Institute of Biotechnology, Amity University, Raipur, Chhattisgarh, India; ^2^Department of Biotechnology and Microbiology, Anjaneya University, Raipur, Chhattisgarh, India; ^3^Department of Food Science and Nutrition, Faculty of Home Economics, Helwan University, Cairo, Egypt; ^4^Department of Food Science and Nutrition, College of Food and Agricultural Sciences, King Saud University, Riyadh, Saudi Arabia; ^5^Department of Animal Biosciences, University of Guelph, Guelph, ON, Canada

**Keywords:** Chhattisgarh, coalfields, melanin, response surface methodology, UV–Vis spectroscopy, FTIR

## Abstract

In the present study, fungi were isolated and screened from barren land in south-eastern Coalfields limited (SECL) in Chhattisgarh, India. Out of 14 isolated fungi, only three fungal isolates exhibited pigmentation in screening studies. The isolated fungal strain SP1 exhibited the highest pigmentation, which was further utilized for *in vivo* production, purification, and characterization of melanin pigment. The physical and chemical properties of the fungal pigment showed insolubility in organic solvents and water, solubility in alkali, precipitation in acid, and decolorization with oxidizing agents. The physiochemical characterization and analytical studies of the extracted pigment using ultraviolet–visible spectroscopy and Fourier transform infrared (FTIR) confirmed it as a melanin pigment. The melanin-producing fungus SP1 was identified as *Thermothelomyces hinnuleus* based on 18S-rRNA sequence analysis. Furthermore, to enhance melanin production, a response surface methodology (RSM) was employed, specifically utilizing the central composite design (CCD). This approach focused on selecting efficient growth as well as progressive yield parameters such as optimal temperature (34.4°C), pH (5.0), and trace element concentration (56.24 mg). By implementing the suggested optimal conditions, the production rate of melanin increased by 62%, resulting in a yield of 28.3 mg/100 mL, which is comparatively higher than the actual yield (17.48 ± 2.19 mg/100 mL). Thus, *T. hinnuleus* SP1 holds great promise as a newly isolated fungal strain that could be used for the industrial production of melanin.

## 1 Introduction

Melanin is a complex natural biopolymer formed through the oxidation of phenols, followed by the polymerization of intermediate phenols and the resulting quinones. It can be classified into three main types based on its structural components: eumelanin, pheomelanin, and allomelanin. Eumelanin and allomelanin are responsible for imparting dark brown coloration to cells, while pheomelanin, found in the animal kingdom, contributes to yellow or red pigmentation (Suryanarayanan and Thennarasan, 2004; Choi, 2021; Singh et al., 2021; Elsayis et al., 2022). Melanin is ubiquitous across various life forms, including microorganisms, plants, and animals, and it serves crucial defensive functions (Suwannarach et al., 2019; Ghadge et al., 2021; El-Naggar and Saber, 2022). One of its primary roles is photoprotection and shielding against harmful UV radiation. Additionally, melanin functions as a potent scavenger of free radicals, possesses metal ion chelation capabilities, and contributes to heat and drought tolerance (Cordero and Casadevall, 2017; El-Naggar and Saber, 2022; Suthar et al., 2023). It also plays a part in maintaining cell wall rigidity and has the capacity to absorb heavy metals (Tran-Ly et al., 2020; Singh et al., 2021). These diverse physiochemical properties render melanin suitable for a wide array of applications in bio-electrochemistry, including its use as a UV absorber, cation exchanger, drug carrier, and amorphous semiconductor (Ye et al., 2014; Ghadge et al., 2021). Fungal melanin has also been shown to absorb non-ionizing radiation for heat capture. Moreover, fungal melanin protects yeast from spaceflight effects (Dadachova and Casadevall, 2008; Cordero et al., 2018; El-Bialy et al., 2019). In the derma-cosmetic industry, melanin is extensively used due to its UV-protective properties commonly applied in the production of sunscreen and related products (Choi, 2021; El-Naggar and Saber, 2022). Moreover, melanin has demonstrated impressive biological activities, including anti-cancer, antimicrobial, anti-inflammatory, and hepatoprotective properties (El-Naggar and El-Ewasy, 2017; Huang et al., 2018; Ghadge et al., 2021; Surendirakumar et al., 2022).

Due to the growing demand for melanin pigment, there is a pressing need for research on its production and the discovery of new sources (Elsayis et al., 2022). Traditionally, melanin has been derived from *Sepia officinalis* extract or produced synthetically (Srisuk et al., 2016; Pralea et al., 2019). However, these conventional methods come with drawbacks, including high production costs, limited flexibility, and environmental pollution risk (Choi, 2021; El-Naggar and Saber, 2022). Consequently, there has been significant interest in exploring alternative sources of melanin through microbial bioproduction. Various microorganisms, such as bacteria, actinomycetes, and fungi, naturally produce melanin as a response to environmental stresses (Suryanarayanan and Thennarasan, 2004; Gessler et al., 2014; Elsayis et al., 2022). Fungi, in particular, are known to produce melanin in adverse conditions and can serve as a natural sunscreen, offering protection against UV radiation. Melanin plays a crucial role in helping fungi survive in environments with intense UV exposure. Furthermore, melanin possesses strong anti-oxidative properties that safeguard fungal cells from oxidative stress, including that induced by reactive oxygen species (Nosanchuk and Casadevall, 2006; Zhan et al., 2011; Singh et al., 2021). In certain fungi, melanin also contributes to maintaining the structural integrity of the cell wall (Nosanchuk et al., 2015). Several studies have investigated the extraction of melanin from various fungal genera, including *Aspergillus*, *Exophiala*, *Gliocephalotrichum*, *Hortea*, *Inonotus*, *Penicillium*, *Phoma*, *Phyllosticta*, *Pleurotus*, *Schizophyllum*, and *Spissiomyces* (Suryanarayanan and Thennarasan, 2004; Selvakumar et al., 2008; Zhan et al., 2011; Jalmi et al., 2012; Kejžar et al., 2013; Liu et al., 2014; Pal et al., 2014; Arun et al., 2015; Hou et al., 2019; Suwannarach et al., 2019; Elsayis et al., 2022; Surendirakumar et al., 2022). However, the yield from these sources remains insufficient to meet the demands of large-scale applications. Consequently, there is an urgent need to isolate microorganisms with high melanin-producing capabilities and to explore methods for enhancing microbial melanin production (Tran-Ly et al., 2020; Choi, 2021).

Coal is the most abundant fossil fuel resource in India, and the country ranks as the third-largest global coal producer, boasting the fourth-largest coal reserves worldwide. Among Indian states, Chhattisgarh is renowned for its mineral wealth and possesses the third largest coal reserve in the country, accounting for 74,191.76 million tons, following Jharkhand and Odisha (Ministry of Coal, Government of India, 2022). In the unique ecosystem of coalfields, air serves as the primary source of fungal colonization, with fungi adapting to exploit both organic and inorganic substances present in their surroundings. Previous reports have documented the presence of airborne fungal species such as *Aspergillus* spp., *Penicillium* spp., *Cladosporium* spp., and *Chaetomium* spp., in Indian coalfields. Thus, fungi can exist in cold fields as either resting propagules or active mycelia, depending on nutrient availability and favorable environmental conditions, making their spores easily detectable in soil samples (Tulsiyan et al., 2017). Exploration of the soil microflora in the coalfields of Chhattisgarh in India for melanin production is an unexplored area with no previous studies carried out. Therefore, the objectives of the present study were to isolate and identify melanin-producing fungi from soil samples collected from the SECL, Bilaspur, Chhattisgarh, followed by the extraction of the melanin pigment from the selected soil fungus, to characterize the melanin pigment by physiochemical analysis, ultraviolet, and FTIR spectroscopy, and to optimize the culture conditions for enhanced melanin production using CCD under *in vitro* conditions. To the best of our knowledge, this is the first study exploring the potential of the soil fungus *T. hinnuleus* SP1 isolated from the SECL in Bilaspur, Chhattisgarh, for producing melanin pigment.

## 2 Materials and methods

### 2.1 Sample collection, isolation, and identification of fungal isolates

Soil samples were collected from the SECL in Bilaspur, Chhattisgarh (22.08 °N, 82.14 ° E), India, in April 2022. The collected soil sample was serially diluted (), and fungi were isolated by plating the diluted soil on potato dextrose agar medium in 90-mm Petri dishes, supplemented with 0.01% w/v chloramphenicol (PDAC). Plates were sealed with double parafilm and incubated at 28 ± 1°C for 14 days under dark conditions. The incubated plates were regularly checked (every 48 h) for fungal growth. The growing fungi were further sub-cultured onto a fresh PDAC. Furthermore, only representative isolates were maintained in glycerol stock (40%) as an axenic culture for further use, and repetitive isolates were discarded (Karkun Sur et al., 2016; Karkun Sur and Verma, 2016). Furthermore, for morphological identification (up to genus level) of the isolated fungi, morphological structures such as colony color, texture, pigment production, and microscopic characters such as hyphae, conidia, stipe, and phialides were observed and recorded. Based on the observed morphological and microscopic characters, fungi were identified following standard mycological keys (Barnett and Hunter, 1998).

### 2.2 Preliminary assessment of melanin production

Melanin production by isolated fungal species was assessed by following the previously described methods with some minor modifications. Briefly, an actively growing mycelial plug (5 mm) of 7-day-old fungal culture was inoculated onto L-tyrosine (2%) PDA medium, sealed with double parafilm, and incubated in dark conditions for 14 days. Fungal isolates exhibiting black pigmentation were selected and sub-cultured for up to three generations to check the stability of the pigmentation. A fungal isolate that exhibited stability and maximum pigmentation was selected for further identification and characterization (Kejžar et al., 2013).

### 2.3 Molecular identification of melanin-producing fungus

For the molecular identification of the melanin-producing fungus, about 0.5 to 1 g of 7-day-old fungal mycelia was crushed to a very fine powder in a pestle and mortar using liquid nitrogen. The powdered mycelia were then transferred to a microcentrifuge tube, and DNA extraction was performed using the spin column kit (HiMedia, India) following the manufacturer’s instructions. Further, the evolutionary relationship was established by amplifying the 18S-rRNA gene (1,000 bp) (White et al., 1990) using a polymerase chain reaction in a thermal cycler and was purified using exonuclease I – shrimp alkaline phosphatase treatment (Darby et al., 2005). Purified amplicons were sequenced by the Sanger method in an ABI 3500xl genetic analyzer (Life Technologies, United States) at the National Collection of Industrial Microorganisms, CSIR-National Chemical Laboratory, Pune, India. The obtained sequences were edited using CHROMASLITE (version 1.5) and submitted to GenBank under the accession number OR584212. The final consensus sequence was subjected to an NCBI BLAST^1^ similarity search to ascertain putative positional homology with closely related organisms (Altschul et al., 1990).

### 2.4 Cultivation of fungal mycelium and pigment production

A liquid culture of potato dextrose broth was prepared by growing fungus in pre-sterilized potato dextrose broth (PDB). Concisely, the method consisted of the aseptic inoculation of 100 mL of pre-sterilized PDB in a 250 mL Erlenmeyer flask (Borosil, India) supplemented with L-tyrosine (0.5–2%, w/v) with mycelial plugs (5 mm) of a 7-day-old culture of *Thermothelomyces hinnuleus* S1 and incubated at 28 ± 1°C under static and shaking conditions (130 rpm) for 3 weeks. Following incubation, the color of the broth turned brownish black as the fungal strain started producing pigment (Supplementary Figure S1). After the completion of the incubation period, the fungal biomass was separated from the broth by filtration, followed by centrifugation at 8000 g for 10 min. The obtained fungal mycelium was collected and dried in a hot air oven at 60 ± 1°C for 24 h. The mycelial biomass was further evaluated for melanin production (Singh et al., 2021).

### 2.5 Extraction and partial purification of fungal melanin

The extraction of melanin pigment was carried out by the acid/alkali method (Gadd, 1982; De la Rosa et al., 2017) with some minor modifications. The dried fungal biomass was dipped in a beaker with 1 N KOH solution overnight and autoclaved at 121°C for 20 min. Mycelium was crushed into fine particles by adding a small amount of double-distilled water with the help of a mortar and pestle. The solution was centrifuged at 5000 g for 15 min to collect the supernatant. The obtained supernatant was acidified with a 3 N HCL solution until the pH reached 2.5. The supernatant was precipitated and was again centrifuged at 8000 g for 15 min to collect the pellets (Gadd, 1982).

The obtained pellets were washed three times with double-distilled water and extracted using ethanol, chloroform, and ethyl acetate in a ratio of 2:3:2. The resultant pellet thus obtained was completely dried in a hot air oven at 60°C. The melanin was collected in powdered form. The obtained melanin powder was finally dissolved in a 10% KOH solution (Rajagopal et al., 2011).

### 2.6 Characterization of fungal melanin

#### 2.6.1 Physiochemical characterization

Tests were performed according to the procedure followed by Suwannarach et al. (2019) to characterize the fungal pigment obtained from *T. hinnuleus* SP1. The primary physiochemical properties of the fungal melanin, such as solubility in distilled water, common organic solvents such as acetone, chloroform, dimethyl sulfoxide, ethanol, methanol, ethyl acetate, and inorganic solvents such as 1 M KOH, were determined. Furthermore, H_2_O_2_ and Fecl_3_ were used for bleaching and precipitation tests (Zaidi et al., 2014).

#### 2.6.2 Spectrophotometric analysis

The obtained fungal melanin (1 mg) was dissolved in 10% 1 M KOH (20 mL) following the method described by Rajagopal et al. (2011). The UV–visible absorption spectrum of the fungal melanin was scanned in the wavelength range of 200–420 nm using a UV–visible spectrophotometer (UV–Vis spectrophotometer Evolution™ 201/220) by comparing it with a standard L-DOPA melanin (Sigma Aldrich, St. Louis, MO, United States). The 10% 1 M KOH solution served as a reference blank. The maximum absorption of fungal melanin and standard L-DOPA melanin was recorded.

#### 2.6.3 Fourier transform infrared spectrum analysis

The purified fungal melanin and the standard L-DOPA melanin were homogenized with analytical-grade potassium bromide and processed for FTIR. The samples were pressed into disks under vacuum using a KBr press (Singh et al., 2021). The FTIR spectra were recorded using Benchtop Lt4100 Labtronics. The spectra were read at a resolution of 4 cm^−1^ within the range of 400–4,000 cm^−1^. The FTIR analysis was performed at Macates Research Analysis (a division of Pretty Petals (p) Ltd.) 106–107, HSIIDC, Faridabad, Haryana, India.

### 2.7 Optimization of melanin production by using central composite design

Through a literature survey, the major three parameters affecting the production rate were chosen for analysis: temperature, pH, and trace elements. Each independent positive variable was analyzed at three different levels: low, moderate, and high (−1,0, +1). A total of 20 sets of experiments were run in an experimental design (Surwase et al., 2012). The calculated response was recorded as mg/100 mL. The 3D surface plot was made to study the better interaction of the variables. The optimum medium concentration was determined using a second-order polynomial model:


Y=β0+ΣβiXi+ΣβiiXii+ΣβijXij

where β represents regression coefficients for regression for individual factors, βii represents regression coefficients for factor square effects, and βij represents regression coefficients for factor interactions. Based on the result analysis, the CCD experiment was established (Saleh et al., 2018). Design Expert version 13 of Stat-Ease 360 was used to generate statistical results and graphical reports.

## 3 Results

### 3.1 Isolation of soil fungi

A total of 14 fungal morphotypes belonging to 9 different genera were isolated and identified in the present study. Among them, members of the genera *Aspergillus* were found to be the highest in number, with four species, followed by *Penicillium* and *Talaromyces* (two species each). However, only a single species could be recovered from each genus of *Alternaria*, *Fusarium*, *Curvularia*, *Mucor*, *Trichoderma*, and *Thermothelomyces* (Table 1).

**Table 1:** Isolation and screening of fungal isolates from soil samples collected from South-Eastern coalfields limited (SECL), Bilaspur, Chhattisgarh for melanin pigment production.

S.no	Culture code	Microscopic identification	Pigment production in L-tyrosine-supplemented PDA plates			
			0.5%	1%	1.5%	2%
1.	SP 1	*Thermothelomyces* sp.	+	++	++	+++
2.	SP 2	*Alternaria* sp.	−	+	+	++
3.	SP 3	*Aspergillus* sp.	+	+	++	++
4.	SP 4	*Fusarium* sp.	−	−	−	−
5.	SP 5	*Curvularia* sp.	−	−	−	−
6.	SP 6	*Talaromyces* sp.	−	−	−	−
7.	SP 7	*Talaromyces* sp.	−	−	−	−
8.	SP 8	*Aspergillus* sp.	−	−	−	−
9.	SP 9	*Trichoderma* sp.	−	−	−	−
10.	SP 10	*Aspergillus* sp.	−	−	−	−
11.	SP 11	*Aspergillus* sp.	−	−	−	−
12.	SP 12	*Penicillium* sp.	−	−	−	−
13.	SP 13	*Penicillium* sp.	−	−	−	−
14.	SP 14	*Mucor* sp.	−	−	−	−

(+) sign denotes the intensity of the pigmentation. (+) low pigment production, (++) moderate pigment production, (+++) high pigment production, (−) indicates no pigmentation.

### 3.2 Screening of melanin-producing fungi

**Figure 1:**
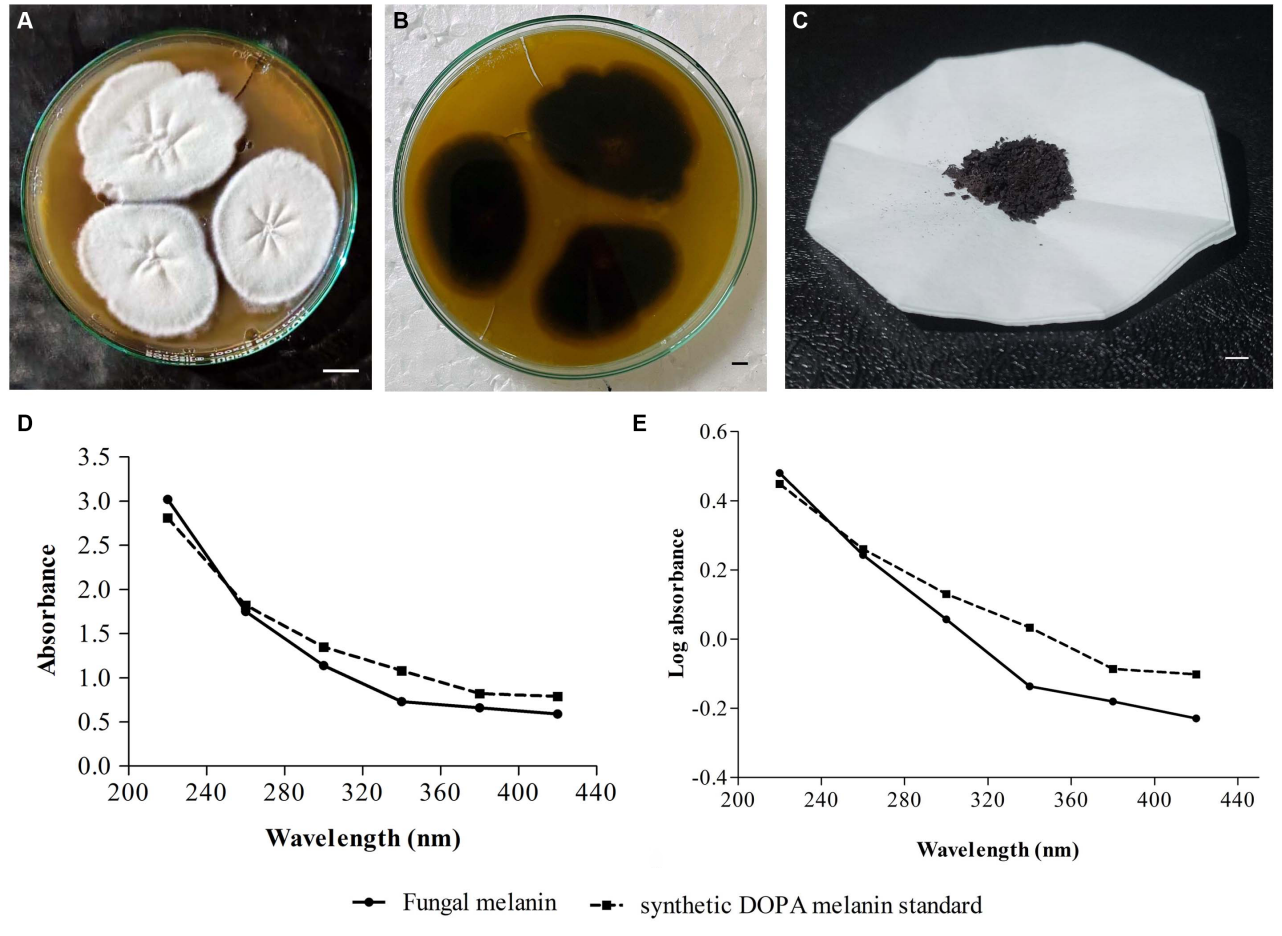
Colonies of *Thermothelomyces hinnuleus* SP1 on potato dextrose agar at 28 ± 1°C, **(A)** colony front view and **(B)** colony reverse view. **(C)** Pellets of fungal melanin after extraction and purification. **(D)** Ultraviolet–visible (UV–visible) absorbance spectrum of pigment from fungal melanin and synthetic DOPA melanin standard. **(E)** A plot of log optical density against wavelength of fungal melanin and synthetic DOPA melanin standard. Bar 10 mm.

In the screening studies, only three fungal isolates (SP1, SP2, and SP3) exhibited black-brown pigmentation on PDA medium supplemented with L-tyrosine. A dose-dependence in pigmentation was observed in all three isolates with maximum pigmentation observed at 2% L-tyrosine. Among the three pigment-producing fungal isolates, *T. hinnuleus* SP1 exhibited the maximum pigmentation (Figures 1A,B), followed by *Aspergillus* sp. SP2 and *Alternaria* sp. SP2, respectively. The other fungal isolates did not produce any pigment under the given conditions (Table 1). Therefore, the fungal strain *T. hinnuleus* SP1 was used for further experiments.

### 3.3 Production of melanin pigment

The fungal strain *T. hinnuleus* SP1 produced a brownish-black mycelial mat with a droplet of diffusible blackish pigment in the PDB broth after 21 days of incubation at 28 ± 1°C. The average yield of crude melanin pigment in *T. hinnuleus* SP1 was 17.48 ± 2.19 mg/100 mL (Figure 1C). Furthermore, higher pigmentation was observed under the shaking condition in comparison to the static condition (data not shown), suggesting that shaking is important for the growth of fungus and melanin production.

### 3.4 Molecular identification and phylogenetic relationship

The fungal strain was identified using 18S-rRNA sequencing. The resultant sequence was compared with the nucleotide sequence present in the NCBI database using the BLAST search program. Based on NCBI-BLAST results, the fungal isolate SP1 (GenBank accession no. OR584212) showed 99.51% identity with *Thermothelomyces hinnuleus* ATCC 52474 (GenBank accession no. NG064994, Query coverage: 100%, E-value: 0). Thus, based on NCBI-BLAST analysis, fungal isolate SP1 was identified as a member of *Thermothelomyces hinnuleus.*

### 3.5 Characterization of fungal melanin

#### 3.5.1 Physiochemical characterization

The physical and chemical properties of fungal pigment and synthetic DOPA melanin standards are shown in Table 2. The fungal pigment and synthetic DOPA melanin standard were insoluble in distilled water, methanol, ethanol, ethyl acetate, chloroform, and acetone. However, the fungal pigment and the synthetic DOPA melanin standard exhibited partial solubility in DMSO and complete solubility in 1 M KOH. Both fungal pigment and the DOPA melanin standard displayed precipitation in 1% FeCl_3_. Both the fungal pigment and synthetic DOPA melanin were positive for decolorization by 30% H_2_O_2_. The physiochemical properties of *T. hinnuleus* SP1 were similar to those of the synthetic DOPA melanin standard.

**Table 2:** Physiochemical properties of the extracted fungal melanin pigment from *Thermothelomyces hinnuleus* SP1 and standard DOPA melanin standard.

S.no.	Properties	Observations	
		Fungal melanin	DOPA melanin standard
1.	Color observation	Blackish brown	Blackish brown
2.	Solubility test		
a.	Methanol	Insoluble	Insoluble
b.	Ethanol	Insoluble	Insoluble
c.	Ethyl acetate	Insoluble	Insoluble
d.	Acetone	Insoluble	Insoluble
e.	Chloroform	Insoluble	Insoluble
f.	Distilled water	Insoluble	Insoluble
g.	DMSO	Partially soluble	Partially soluble
h.	1 M KOH	Soluble	Soluble
3	Precipitation		
a.	1% FeCl_3_	Precipitated	Precipitated
4.	Oxidizing agent		
a.	30% H_2_0_2_	Decolorized	Decolorized

#### 3.5.2 Spectrophotometric characterization

The fungal pigment, in comparison to the synthetic DOPA melanin standard, was spectrophotometrically analyzed in a wavelength range of 220–420 nm. A similar UV–visible absorption spectrum pattern was obtained between both the fungal pigment and the synthetic DOPA melanin standard, exhibiting that the absorption peak was in the UV region and declined toward the visible region with maximum absorption at 220 nm (Figure 1D). Furthermore, when the log optical density against wavelengths of fungal melanin and synthetic DOPA melanin standards were plotted, an almost linear curve with a negative slope was observed (Figure 1E).

#### 3.5.3 Fourier transform infrared spectrum analysis

**Figure 2:**
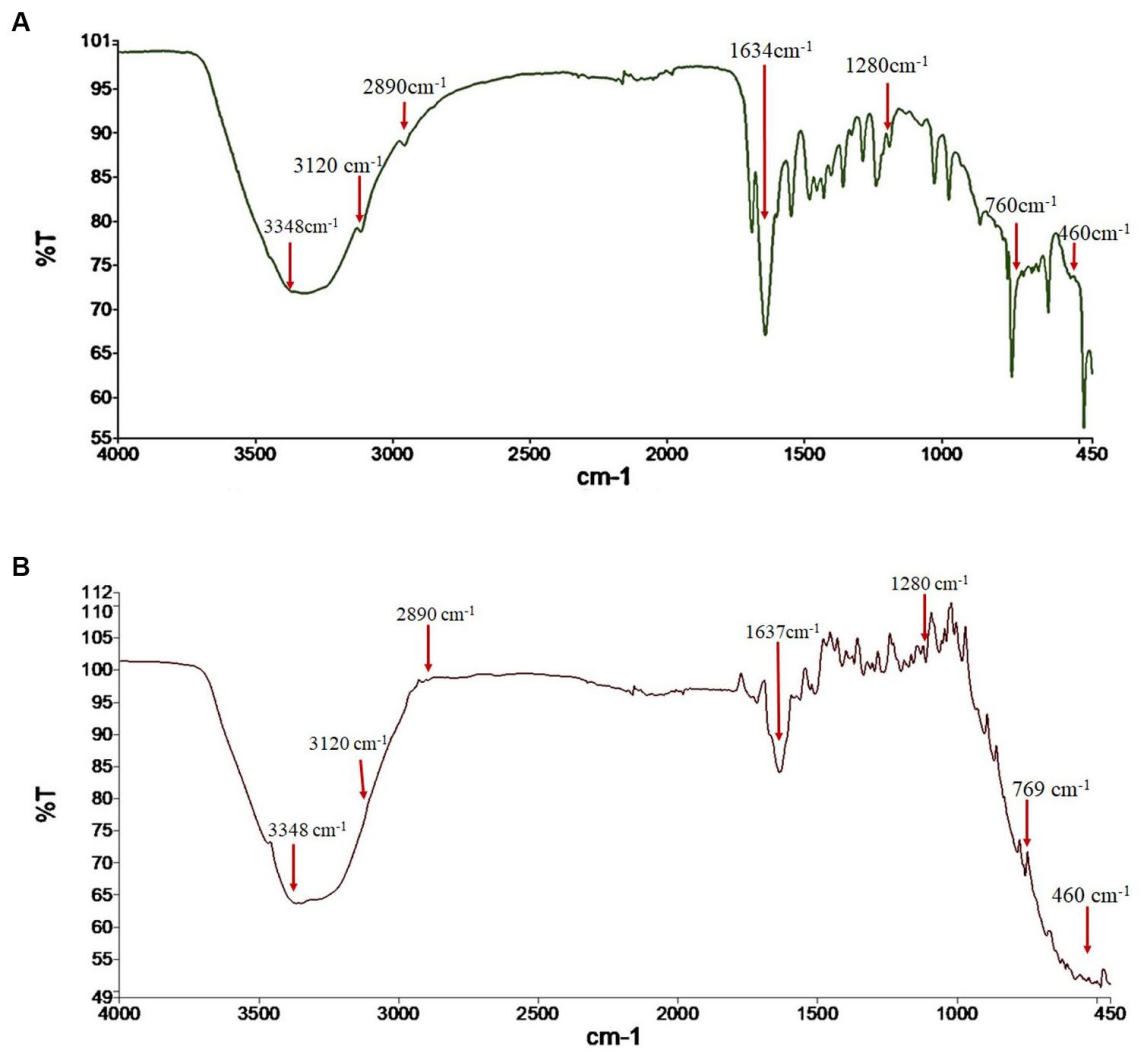
Fourier transform-infrared (FTIR) spectrum analysis of **(A)** fungal melanin from *Thermothelomyces hinnuleus* SP1 and **(B)** synthetic DOPA melanin standard.

The FTIR spectra were analyzed to verify the identity of the fungal pigment as melanin. The FTIR of both the extracted fungal pigment and the synthetic DOPA melanin standard is shown in Figure 2. The obtained data of the fungal pigment showed peaks near 3,348 cm^−1^ showing OH-stretching and aliphatic primary amines, 3,120 cm^−1^ showing CH-stretching, 2,890 cm^−1^ showing NH-stretching, 1,634 cm^−1^ showing C=C stretching, 1,280 cm^−1^ showing C-O stretching, 760 cm^−1^ showing C-H bending, and 460 cm^−1^ showing C-I stretching (Figure 2A). The data for synthetic melanin showed similar peaks with some noticeable differences at 1634 cm^−1^ and 760 cm^−1^ (Figure 2B).

### 3.6 Optimization of melanin production by using central composite design

Three independent variables that are most likely to affect the production rate were analyzed in this experiment using CCD. The matrix of the design and response yield (mg/ml) of each trial were described (Table 3). The determination coefficient R2 for melanin yield is 0.911, showing a desirable relationship between the experiment and the predicted value. The coefficient estimate represents the expected change in response per unit change in factor value when all remaining factors are held constant. The intercept in an orthogonal design is the overall average response of all the runs. The coefficients are adjustments around that average based on the factor settings. The *p*-value is less than 0.05, which shows the model is significant.

**Table 3:** Transforming design matrix into coded representation and expressing responses in actual form.

Run	Factor 1 (Temperature °C)	Factor 2 (pH)	Factor 3 (KCl)	Response (mg/100 mL)
1	0	0	0	52.01
2	0	0	0	50.23
3	-1	1	1	38
4	1	1	1	3.06
5	-1	1	-1	30.45
6	−1.31	0	0	2.5
7	0	1.31	0	55.03
8	−1	−1	1	24.06
9	0	0	0	52
10	0	0	0	51.006
11	0	0	−1.31	20.29
12	1	1	−1.	1.08
13	1	−1	−1	0.38
14	1	−1	1	1.3
15	1.31	0	0	0
16	−1	−1	−1	19.78
17	0	0	0	51
18	0	0	0	51.43
19	0	0	1.31	35
20	0	−1.31	0	25.89

**Figure 3:**
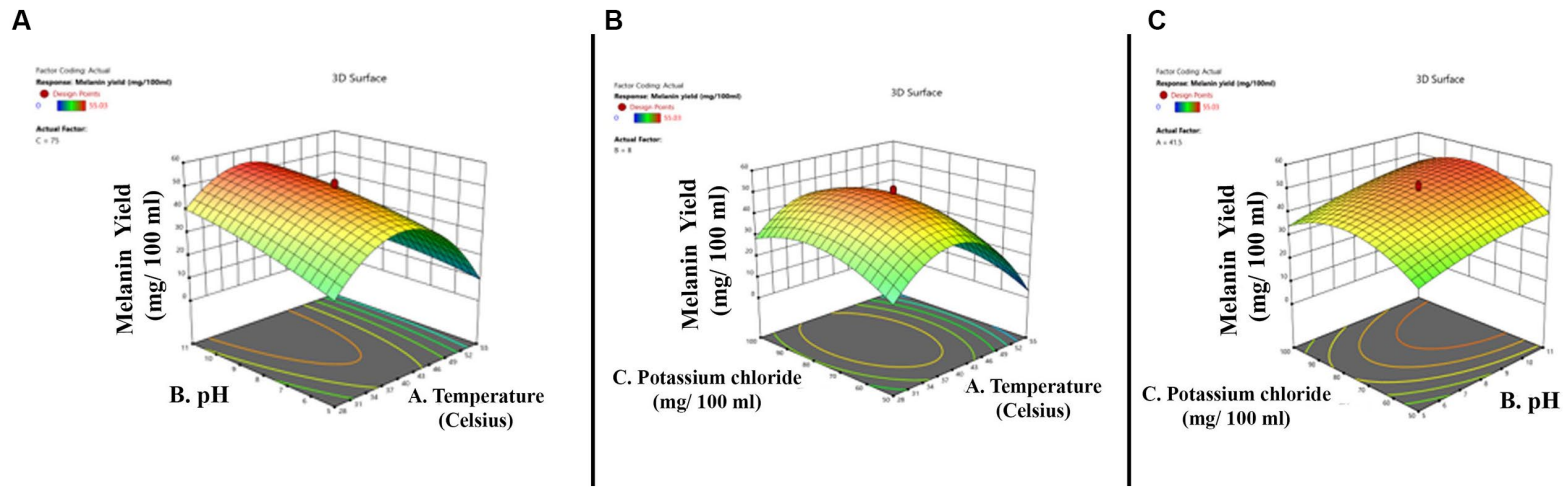
Three-dimensional quadratic model shows the relationship between the tested independent variables and pigment formation by *Thermothelomyces hinnuleus* SP1 and the optimal levels of the three factors as obtained from the maximum of the polynomial model. The three figures indicate **(A)** the interaction between pH and temperature **(B)** the interaction between potassium chloride and temperature, and **(C)** the interaction between potassium chloride and pH.

The three-dimensional surface graph was plotted by Design Expert version 11 software to demonstrate the interaction between the independent variables to enhance melanin production (Figures 3A–C). The quadratic model optimum value for melanin production is temperature 34.44°C; pH 5.0; and potassium chloride 56.24 mg. The optimum conditions were put forward, and experimental validations showed the melanin production yield is 28.32 mg/100 mL, and the model’s predicted value is 30.42 mg/100 mL, which is slightly higher than the actual yield.


$$\begin{array}{l} Y=49.19-9.57\;A+5.71\;B+2.97\;C-2.77\;\mathrm{AB}\\ -1.12\;\mathrm{AC}+0.5413\;BC-24.81\;A^{2}-2.81\;B^{2}-9.58\;C^{2} \end{array}$$

From the above-mentioned data, it can be predicted that for melanin production from the fungal strain *T. hinnuleus* SP1, the conditions can be adjusted. The fungal culture can be grown in a 2% L-tyrosine supplement in a shaking condition at 130 rpm for 3 weeks in a dark environment. The temperature should be 34°C, the pH should be 5, and potassium chloride should be added to the medium as 56.24 mg/100 mL of stock solution. The above optimization method helped in increasing the yield of fungal melanin by 61.8%.

## 4 Discussion

Melanin is a valuable bioactive compound with potential applications in biotechnological, environmental, and pharmaceutical fields. These include their usage in (but not limited to) cosmetics, free radical scavenging, drug delivery, antimicrobials, cancer therapy, and the absorption of heavy metals (Choi, 2021; Singh et al., 2021). The conventional methods for melanin production fall short in fulfilling global demand. Hence, researchers are exploring novel avenues for large-scale production of melanin (El-Naggar and Saber, 2022). Microorganisms are proposed as a sustainable and cost-effective bio-resource for melanin production. Microorganisms from various ecological niches have been investigated for melanin production (Tran-Ly et al., 2020; Singh et al., 2021). In the present study, we investigated soil fungi isolated from barren land near coal mines as a novel source of melanin. Fungal species such as *Aspergillus*, *Penicillium*, and *Curvularia* isolated in the present study were also reported from the coal mines of Hazaribagh, Jharkhand, India (Tulsiyan et al., 2017). Out of 14 screened fungi, only 3 fungal isolates, *viz.*, *T. hinnuleus* SP1, *Alternaria* sp. SP2, and *Aspergillus* sp. SP3, exhibited stable pigmentation. Our findings are in accordance with previous studies, which suggested *Aspergillus* and *Alternaria* spp. as prolific producers of melanin pigment (Pal et al., 2014; El-Batal and Al-Tamie, 2016; Fernandes et al., 2021). To the best of our knowledge, this is the first report of melanin pigment extracted from the soil fungus *T. hinnuleus* SP1. The fungal strain *T. hinnuleus* SP 1 isolated in the present study has been documented as a rare thermophilic fungus and was previously isolated from soil samples in Japan and New Zealand. *Thermothelomyces hinnuleus* is the only species in its genus that produces verrucose, pigmented conidia (Marin-Felix et al., 2015; Wang et al., 2022). Moreover, for accurate identification of the fungal species, we sequenced the 18S-rRNA region of the fungus. The 18S-rRNA sequence analysis indicated a 99% similarity of the melanin-producing isolate SP1 with *T. hinnuleus* ATCC 52474. The sequence similarity of the 18S-rRNA region has been previously used for the identification of *Thermothelomyces* because of its significant variability among organisms (Hou et al., 2021).

The fungal strain *T. hinnuleus* SP1 produced melanin pigment in both fungal biomass and its culture broth, whereas the melanin was ultimately extracted from fungal biomass. The obtained results were similar to those of previous studies, which observed that filamentous fungi such as *Alternaria alternata*, *Aspergillus oryzae*, *A. sydowii*, *A. terreus*, *Fusarium oxysporum*, *Exophiala pisciphila*, *Penicillium* spp., *Phoma* sp. RDSE17, and *Spissiomyces endophytica* exhibited melanin production after being cultivated either on solid or liquid media (Zhan et al., 2011; Pal et al., 2014; El-Batal and Al-Tamie, 2016; Suwannarach et al., 2019; Fernandes et al., 2021; Surendirakumar et al., 2022). Numerous studies have examined the synthesis of melanin in cultured fungal biomass of various fungal species (Kumar et al., 2011; Zhan et al., 2011; Pal et al., 2014; Xu et al., 2020) or using submerged cultivation techniques in PDB or similar growth media (Selvakumar et al., 2008; El-Naggar and El-Ewasy, 2017; Elsayis et al., 2022). In the present study, the yield of crude pigment (17.48 mg/100 mL) from *T. hinnuleus* SP1 was lower than those reported by Suwannarach et al. (2019) in *S. endophytica* (315 mg/L), Elsayis et al. (2022) in *Hortaea werneckii* AS1 (420 mg/L), and Surendirakumar et al. (2022) in *Phoma* sp. RDSE17 (540 mg/L). In contrast, the melanin production in *T. hinnuleus* SP1 was higher than that reported in *Yarrowia lipolytica* (16 mg/L) and *E. pisciphila* (12 mg/L) (Zhan et al., 2011; Tahar et al., 2019). Variation in the amount of melanin produced can primarily be attributed to differences among species and even among different strains within a species when it comes to their natural pigment production. While the techniques for the extraction and the type of cultivation media and conditions used are also significant factors, it is widely recognized that certain fungi inherently produce a higher amount of pigment compared to other species (Suwannarach et al., 2019; Surendirakumar et al., 2022). It was also observed that pigmentation was higher under shaking conditions as compared to static conditions. The reason why agitation has a direct impact on the productivity of melanin in *T. hinnuleus* SP1 can be attributed to the fact that the majority of aerobic microorganism activities are influenced by how quickly oxygen is transferred from the air to the liquid medium. Therefore, the frequency of agitation plays a crucial role in enhancing the circulation of oxygen, which in turn affects both the growth of the microorganism and its pigment production (Hermann et al., 2001). Moreover, it is important to emphasize that metal ions play a crucial role in the synthesis of melanin. Zhou et al. (2023) recently revealed that the supplementation of KCl and MgSO_4_ stimulates melanin production and is essential for the proper functioning of enzymes involved in melanin synthesis.

Melanin is composed of different types of carbon–carbon monomer units, which makes their systemic characterizations difficult (Selvakumar et al., 2008). The structure of melanin is poorly understood, and an accurate definition of melanin is still obscure. However, the following criteria are indicative of fungal melanin (Zhan et al., 2011): the black-brown color, insolubility in water and organic solvents, resistance to degradation by acids, concentration by alkali solution, and decolorization by an oxidizing agent. The solubility properties of the extracted fungal melanin from *T. hinnuleus* SP1 were similar to the synthetic DOPA melanin standard, as were the properties of the melanin produced by previously reported microorganisms (Selvakumar et al., 2008; Suwannarach et al., 2019; Elsayis et al., 2022; Surendirakumar et al., 2022). In accordance with our findings, melanin produced by endophytic fungus *S. endophytica* SDBR-CMU319, marine fungus *Nocardiopsis* spp., and actinomycete *Streptomyces glaucescens* NEAE-H were found to be insoluble in water and ethyl acetate and chloroform but was partially soluble in DMSO and KOH solution (El-Naggar and El-Ewasy, 2017; Kamarudheen et al., 2019; Elsayis et al., 2022).

Previous studies on the UV properties of melanin indicate that the optimal absorption wavelength in alkaline solutions varies among different types of melanin and typically falls within the 190–300 nm range. This absorption behavior appears to be influenced by the origin of melanin. Furthermore, melanin solution in alkaline environments displays significant absorbance in the high UV region, which gradually declines toward the visible region. This behavior of melanin in an alkaline solution is likely attributed to the complex structures of its conjugated molecules, which interact and scatter UV light photons. Fungal melanin produced by *T. hinnuleus* SP1 showed its highest peak at 220 nm, which was consistent with the UV spectra of melanins from various microbial sources, including actinomycetes, bacteria, and fungi. Melanin produced by fungal species such as *E. pisciphila*, *S. endophytica* SDBR-CMU319, *Phoma* sp. RDSE17, and *P. cystidiosus* var. *formosensis* exhibited similar characteristic peaks with maximum absorbance in the UV region detected in the range of 216–280 nm (Selvakumar et al., 2008; Zhan et al., 2011; Suwannarach et al., 2019; Surendirakumar et al., 2022). Following a similar pattern, melanin produced by *H. werneckii* AS1 also showed maximum absorbance at 240 nm, whereas the standard melanin exhibited the highest absorbance at 219 nm (Elsayis et al., 2022).

FTIR spectroscopy is one of the major analytical tools preferred for analyzing the spectral makeup of functional groups in a sample by associating them with their corresponding absorption bands (Surendirakumar et al., 2022). The obtained FTIR absorption spectra of *T. hinnuleus* SP1 revealed a strong peak near 3,348, 3,120, 2,890, 1,634, and 1,280 cm^−1^, indicating the presence of hydroxyl (-OH and –NH) and aromatic (C=C, CH, and COO) groups. The FTIR spectrum of the fungal melanin exhibited a broad absorption feature, indicating the existence of hydrogen bonding involving the OH group and aromatic ring C=C stretching. These absorption peaks correlated with those identified in synthetic melanin standards and melanin pigments found in various fungal species found in previous studies (El-Naggar and El-Ewasy, 2017; Suwannarach et al., 2019; Elsayis et al., 2022; Surendirakumar et al., 2022).

Response surface methodology has been utilized as an insightful statistical technique for optimizing melanin production from 17.48 to 28.3 mg/100 mL, boasting an increase of approximately 62%. Previous studies utilizing the RSM for optimizing melanin production in fungi have significantly contributed to our understanding of this process. This design helps in understanding the optimal conditions affecting production rates. The melanin production by *A. fumigatus* increased twofold, whereas in the case of *Brevundimonas* sp. SGJ, the melanin production increased 3.05 times (Surwase et al., 2012; Raman et al., 2015). Furthermore, studies carried out by Elsayis et al. (2022) also highlighted the efficiency of the RSM technique for improving melanin yield. These studies provided a framework for systematically investigating the factors influencing melanin production and for identifying optimal conditions. The insights gained from these studies have far-reaching implications, enabling the high-yield production of melanin from *T. hinnuleus* SP1 isolated from barren soil of the SECL, Bilaspur, Chhattisgarh, India.

## 5 Conclusion

Melanin pigment was synthesized and extracted from the fungus *T. hinnuleus* SP1, which was isolated from the SECL region. Physiochemical characterization and analytical studies of the extracted pigment using UV–visible spectroscopy and FTIR confirmed it to be a melanin pigment. The optimized production of melanin was obtained using RSM at a temperature of 34.4°C when the pH was 5.0 and the trace element concentration was 56.24 mg. These findings have the potential to significantly enhance melanin yield, making them a valuable contribution to scale-up production and various applications in cosmetics, pharmaceuticals, and materials science. Overall, the results collectively emphasize the potential of *T. hinnuleus* SP1 as an efficient melanin producer and highlight its significance in various fields. The study opens up avenues for further research to explore the biological activities and industrial applications of the melanin pigment. Investigating the genetic basis of melanin synthesis in this strain could pave the way for bioengineering and biotechnological advancements in melanin production. In summary, this comprehensive study provides a solid foundation for understanding and harnessing the capabilities of *T. hinnuleus* SP1 in melanin production and its potential applications.

## Data availability statement

The datasets presented in this study can be found in online repositories. The names of the repository/repositories and accession number(s) can be found at: https://www.ncbi.nlm.nih.gov/genbank/, OR584212.

## Author contributions

SP: Conceptualization, Data curation, Formal analysis, Investigation, Methodology, Writing – original draft, Writing – review & editing. VM: Conceptualization, Data curation, Formal analysis, Investigation, Methodology, Software, Supervision, Validation, Writing – original draft, Writing – review & editing. HY: Conceptualization, Data curation, Formal analysis, Funding acquisition, Investigation, Methodology, Writing – review & editing. AA: Conceptualization, Data curation, Formal analysis, Funding acquisition, Investigation, Methodology, Writing – original draft, Writing – review & editing. NA: Conceptualization, Data curation, Formal analysis, Investigation, Writing – original draft, Writing – review & editing. AS: Conceptualization, Data curation, Formal analysis, Investigation, Methodology, Project administration, Resources, Software, Supervision, Validation, Writing – original draft, Writing – review & editing.

## Footnotes

^1^ https://www.Ncbi.nlm.Nih.gov/BLAST
