# Supplementary figures and images for: Efficient production and characterization of melanin from Thermothelomyces hinnuleus SP1, isolated from the coal mines of Chhattisgarh, India
Source: Front Microbiol. 2024 Jan 12;14:1320116. doi: 10.3389/fmicb.2023.1320116 (PMC10826702; doi:10.3389/fmicb.2023.1320116)

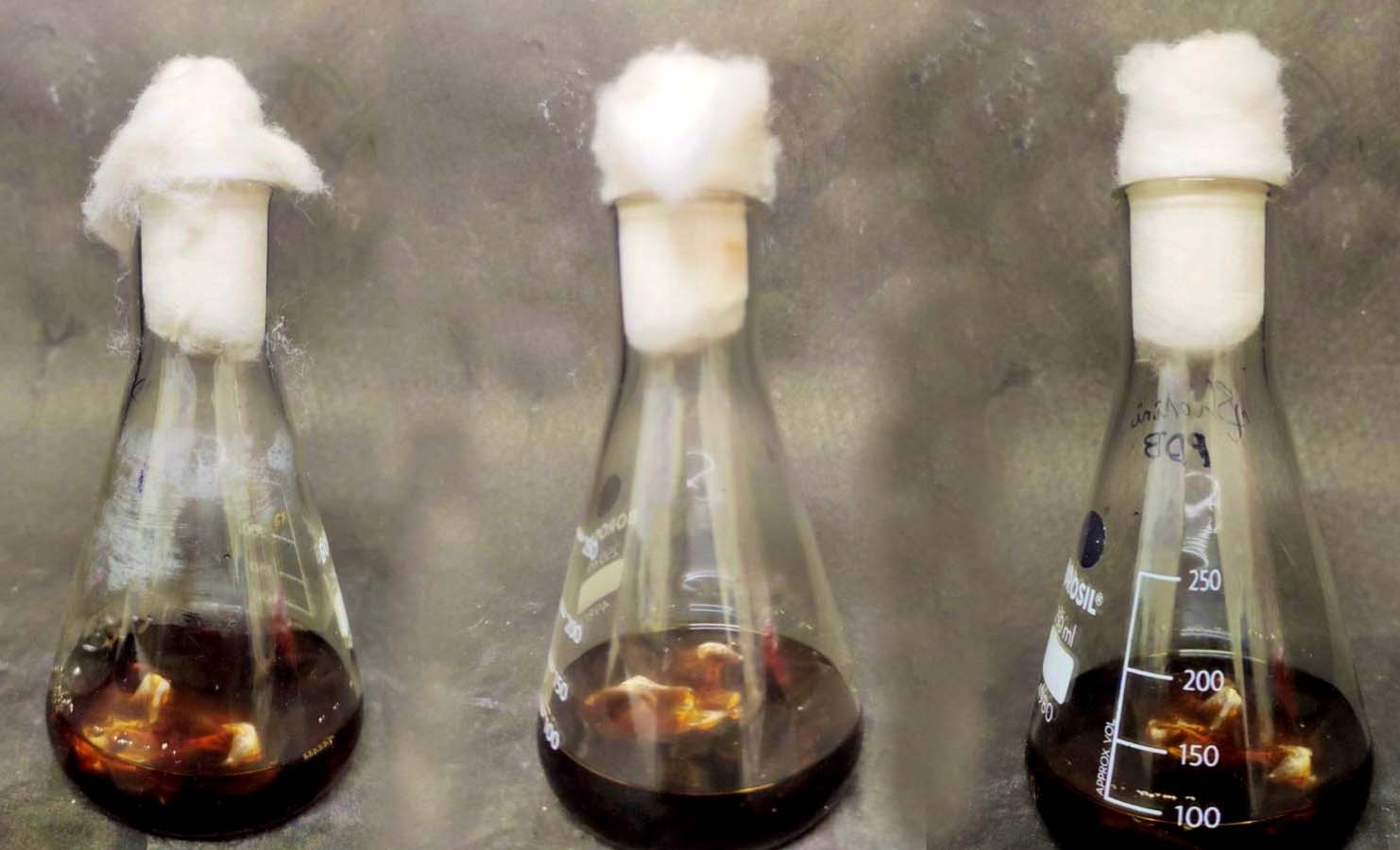

Supplement: Supplementary file 1 [file Image_1.JPEG]
